# From Burnout to Occupational Depression: Recent Developments in Research on Job-Related Distress and Occupational Health

**DOI:** 10.3389/fpubh.2021.796401

**Published:** 2021-12-10

**Authors:** Irvin Sam Schonfeld, Renzo Bianchi

**Affiliations:** ^1^Department of Psychology, The City College and the Graduate Center of the City University of New York, New York, NY, United States; ^2^Institute of Work and Organizational Psychology, University of Neuchâtel, Neuchâtel, Switzerland

**Keywords:** depression, anxiety, occupational depression inventory, work stress and burnout, job-related distress, occupational health science, Maslach Burnout Inventory

## Abstract

Job-related distress has been a focal concern in occupational health science. Job-related distress has a well-documented health-damaging and life-threatening character, not to mention its economic cost. In this article, we review recent developments in research on job-related distress and examine ongoing changes in how job-related distress is conceptualized and assessed. By adopting an approach that is theoretically, empirically, and clinically informed, we demonstrate how the construct of burnout and its measures, long favored in research on job-related distress, have proved to be problematic. We underline a new recommendation for addressing job-related distress within the long-established framework of depression research. In so doing, we present the Occupational Depression Inventory, a recently developed instrument devised to assess depressive symptoms that individuals specifically attribute to their work. We close our paper by laying out the advantages of a paradigm shift from burnout to occupational depression.

## Introduction

Job-related distress, a focal concern of occupational health science, has well-documented health-damaging and life-threatening effects, not to mention economic costs. Burnout and depression have constituted two key indicators in research on job-related distress over the last decades.

Considerable evidence has accumulated to show that chronic exposure to adverse working conditions contributes to the emergence of depressive symptoms and disorders (1–3). Depression can culminate in suicide, including workplace suicide (4). Burnout is thought to reflect the personal impact of chronic exposure to adverse working conditions. Given the common origins of burnout and job-related depressive symptoms and disorders, it is important to examine the evidence bearing on their conceptualization.

In theory, burnout is a gauge of job-related distress. It consists of three symptom dimensions, the core dimension being (emotional) exhaustion (5). Maslach et al. (6) wrote that “[f]or use in applied settings, a prudent approach when deciding to take action on the basis of burnout scores is to give the most weight to Emotional Exhaustion [EE] scores as they are the most reliable” (p. 3), underlining the dimension's central position in burnout. EE refers to feeling emotionally drained—the result of the worker's chronic exposure to adverse job conditions. Burnout's two other dimensions are depersonalization/cynicism (DP) and a reduced sense of personal accomplishment (rPA), also known as professional inefficacy (6). DP refers to the worker being socially distant, disengaged, and possessing a cynical attitude toward coworkers and the people the worker is supposed to serve (e.g., patients, students, customers); rPA refers to the worker feeling that he or she fails to accomplish worthwhile job-related goals. Many researchers have advanced the view that burnout is distinct from depression (5, 7).

This paper has a twofold aim. First, we examine sets of research findings and observations that bear on the conceptualization of job-related distress in the context of the burnout–depression relationship, approached both dimensionally and categorically. Second, we provide a recommendation for assessing job-related distress by way of a new measure that may help occupational health specialists support individuals and organizations more effectively.

## Burnout and Depression

Burnout has been conceptualized as a set of symptoms, largely fatigue-related, that are caused by adverse working conditions. An exemplary item from the most frequently used burnout measure, the Maslach Burnout Inventory (MBI), demonstrates that idea: “I feel emotionally drained from my work” (6). The symptom in question is explicitly attributed to the individual's job. Burnout scales like the MBI have been linked to prominent occupational risk factors such as excessive workloads and reduced control over job tasks (8, 9). From a methodological standpoint, given that burnout items reference both the dependent variable (the symptoms) and independent variable (workplace stressors), it is no surprise that burnout scales are related to perceived workplace stressors, particularly when cross-sectional designs are used [see (10)].

Depression involves a cluster of affective, cognitive, behavioral, and somatic symptoms (e.g., dysphoric mood and anhedonia). Though depression is nosologically and diagnostically defined, there is robust evidence that depression is better conceived of as a dimensional phenomenon—a continuum—with only individuals at the highest end of the continuum meeting criteria for formal diagnoses of depression (11–13). Depressive symptom scales differ from burnout scales in an important way. Except for the recently developed Occupational Depression Inventory (14), described later, depressive symptom scales are “cause-neutral.” This is not to say that depressive symptoms and disorders are void of causes, only that assessment instruments tend to focus on symptom severity rather than symptom causes. Situations involving unresolvable stress, in which individuals feel helpless and trapped in the face of negative events perceived as uncontrollable and impossible to surmount, have long been identified as crucial depressogenic factors (15, 16).

## Observations Bearing on Burnout's Relation to Depression

We now examine six sets of observations that bear on the problematic nature of the burnout construct, particularly regarding its relation to depression. First, although multiple factors can give rise to depression, abundant evidence from well-controlled longitudinal studies indicates that workplace stressors are related to increased levels of depressive symptoms (measured by cause-neutral symptom scales) and elevated risk of depressive disorders (2, 3, 17, 18). It is unlikely that job stressors increase the risk of burnout without commensurately increasing the risk of depressive symptoms and disorders (19, 20).

Second, burnout's core dimension, exhaustion, is highly related to depressive symptoms. Although some meta-analytic evidence suggests an average burnout/exhaustion–depression correlation in the 0.50 s (21, 22), other meta-analytic research (11, 23) indicates that the burnout/exhaustion–depression correlation can reach 0.70–0.80 and higher, particularly when measurement error is controlled. Meier and Kim (22) observed that a correlation of 0.50 has been used to advance the view that (a) burnout and depression *do not* overlap and (b) burnout and depression *do* overlap. Correlation coefficients should be understood in context. The research of Wurm et al. (24) provides some of that context. These authors obtained a burnout–depression correlation of 0.52 in a sample of 5,897 physicians, and observed that, compared to an almost-symptom-free reference group, as burnout symptoms increased stepwise from mild to moderate to severe to extremely high levels, the odds ratio for major depression increased dramatically, from 2.99 to 10.14 to 46.84 to 92.78.

Because fatigue and sleep problems are symptoms of depression (25), fatigue-related items are commonly found in depression symptom scales. Maslach and Leiter (7) argued that, because depression scales include fatigue-related symptom items, a high burnout–depression correlation may be a methodological artifact. However, empirical research in which fatigue-related items were stripped out of depressive symptom scales barely changed the correlation (23, 26).

Exploratory structural equation modeling bifactor analyses extract a general factor on which all items can, theoretically, load and specific factors (bifactors) on which items are allowed to cross-load. In several studies (11, 27, 28), depression and exhaustion items primarily loaded on the general factor. The DP and rPA items tended to load more highly on their respective specific factors than on the general factor with exemplary exceptions. For example, the rPA item “I feel very energetic,” which, understandably, reflects the opposite of exhaustion, has a strong negative loading on the general factor and a much weaker loading on the rPA-specific factor.

What construct do depression and burnout's exhaustion (sub)scales measure? Schonfeld et al. (23, 27) advanced the view that the construct is a dimension of psychopathology that can be labeled psychological distress/dysphoria. More than 40 years ago, Dohrenwend et al. (29) found that measures of dread, sadness, anxiety, helplessness/hopelessness, and poor self-esteem correlated about as highly as the scales' reliabilities permitted, suggesting that the scales reflect the same underlying construct. The investigators labeled the construct psychological distress. More recent research on psychopathology has provided evidence for a distress or internalizing dimension reflecting depressive and related (e.g., anxiety) symptoms (12, 27, 30, 31). Structural equation modeling (SEM) evidence indicates that one cohesive distress factor underlies depressive, anxiety, and exhaustion symptom items (27).

Some have argued that burnout (or depression) may mediate the impact of job stressors on depression (or burnout). However, that the discriminant validity of burnout vis-à-vis depression has not been clearly demonstrated undermines the argument. Moreover, such an argument is difficult to articulate when approaching both burnout and depression dimensionally (i.e., as continua).

Third, if burnout is a syndrome comprising EE, DP, and rPA—as indicated by Maslach and colleagues (5, 6)—one would expect the EE, DP, and rPA subscales of the MBI to be more highly correlated with each other than with non-burnout scales. Meta-analytic and SEM evidence (11, 23), however, indicates that EE is more highly related to depressive symptoms than to DP and rPA, undermining the idea that burnout is a (distinct) syndrome (11, 23). In other words, if exhaustion is part of a syndrome, it is part of a depressive syndrome (10, 11).

Interestingly, several methodological factors work *against* finding the magnitude of the EE–depression correlation to be stronger than the correlations among the MBI's subscales, suggesting that the EE–depression link is sturdy. First, the abovementioned “energetic” item on the rPA subscale of the MBI likely increases the magnitude of the EE–rPA correlation. Second, depression symptom items cover the previous 1 or 2 weeks whereas MBI items cover the previous year. Third, the wording of the items in the MBI subscales are substantially similar, each item referencing work, but depression items cover diverse symptoms without a common causal reference. Despite the influence of these methodological factors, the magnitude of the EE–depression correlation is greater than that of the correlations among the MBI subscales.

How are the MBI's other dimensions, DP and rPA, related to depression? They have non-zero correlations with depressive symptoms. DP supposedly reflects a strategy for coping with EE (5, 32). It is reflective of “not caring anymore,” a feature associated with depression (25). rPA is likely to be a long-term consequence of exhaustion (5, 33). It parallels the negative evaluations of one's worth associated with depression (25).

Fourth, there are no clear or consensual diagnostic criteria for burnout. Maslach et al. (6) indicated that the MBI is not a diagnostic tool. That admonition has not stopped researchers from treating burnout diagnostically. Rotenstein et al. (34) found 142 unique definitions of burnout in the literature on physicians. When attempting to treat burnout diagnostically, researchers generally define a case arbitrarily, as an individual with a score above a predetermined cutoff on a burnout (sub)scale. Bianchi, Schonfeld, and colleagues (35, 36) observed that many researchers identify individuals as cases of burnout based on surprisingly low scale scores. Such case-identification procedures fail to distinguish an individual experiencing pervasive distress from an individual confronting a day in which job stress increased but was still within the “normal range.” When Bianchi et al. (37) used an MBI cutoff corresponding to burnout symptoms experienced at least a few times a week (a frequency assumed to represent pervasive distress), they found that 90% of the individuals in the burnout group met criteria for a provisional diagnosis of depression. Schonfeld and Bianchi (20) obtained similar findings using the Shirom-Melamed Burnout Measure. Such findings suggest that if “clinical burnout” (i.e., burnout as a medical diagnosis) were to be defined someday, its differential diagnosis vis-à-vis clinical depression would be impossible, undermining burnout's clinical validity and usefulness (38).

A related set of findings indicates that as burnout symptoms increase, the risk of meeting criteria for a diagnosis of depression increases. This pattern has been replicated across occupational groups, including physician (24), teacher (20, 37), and dentist (39) samples. Ahola et al. (19) went on to observe that “burnout could be used as an equivalent to depressive symptoms in work life” (p. 35).

Fifth, “[o]ften insomnia or fatigue is the presenting complaint” when a depressed individual seeks help from a clinician [(25); p. 162]. An experienced clinician recognizes the disorder. Freudenberger (40), the first investigator to write about burnout in a research journal, observed that the burned-out individual “looks, acts and seems depressed” (p. 161). Exhaustion complaints can mask broader depressive syndromes, especially in male and younger patients. Exhaustion then acts as a metonym for depression.

Sixth, the nomological networks of burnout's exhaustion core and depression parallel each other. Research has, inter alia, identified parallels in burnout/exhaustion's and depression's relationship to the following: cognitive style [e.g., attentional, interpretational, and memory biases, rumination, pessimistic attributions; (41–43)]; anxiety, workplace support, and exposure to nonwork stressful life events and job-related adversity (20, 27); and job satisfaction, illegitimate work tasks, and work-nonwork interference (44).

In this disquisition on burnout and depression, we do *not* advance the view that the burnout construct is identical to the construct of work-related depression. Extensive findings show that burnout's exhaustion core and fatigue-related symptoms are important symptoms of work-related depression, but *not the only* symptoms of work-related depression. The burnout construct captures a depressive phenomenon, but in a truncated manner.

Some depressed workers may represent themselves as “burned out” to reduce stigma. However, the sparse comparative literature on the issue suggests that both conditions are similarly stigmatizing (45). Moreover, the stigma recruiters attach to burnout is a barrier to employment and promotion (46).

We summarize the reasons for identifying burnout symptoms as depressive symptoms. First, job stressors elicit both burnout and depressive symptoms. Second, burnout's core dimension, exhaustion, correlates highly with depressive symptoms. Third, exhaustion correlates more highly with depressive symptoms than with burnout's other putative burnout dimensions, DP and rPA. Fourth, as burnout symptoms increase, the risk of a depressive diagnosis increases. Fifth, often with depression, the presenting complaint is exhaustion, the core symptom of burnout. Sixth, the nomological networks of burnout's exhaustion core and depression closely parallel each other.

Finally, we assert that the symptoms burnout scales assess are *some* of the symptoms that characterize depression, but not all the symptoms that characterize depression. Kasl (47) already observed that a famous burnout scale that “reflects exhaustion (physical, mental, and emotional) … can be seen as a major component of depression” (p. 396). Burnout scales miss important depressive symptoms, such as suicidal thoughts. We, next, report on a new instrument that better fills the role that burnout scales occupy in identifying workers adversely affected by their jobs.

## The Occupational Depression Inventory

Depression contributes heavily to the worldwide burden of disease (48). Unresolvable stress is a primary depressogenic factor. Intractable stressors are often encountered at work. In view of the problem of job-related stressors evoking depressive symptoms in workers, we recently developed the Occupational Depression Inventory [ODI; (14, 49, 50)]. The ODI references the nine symptoms of major depression (25) and incorporates causal attributions to work. From a psychometric and structural standpoint, the ODI is stronger than burnout scales (14, 49, 50). In addition, the ODI assesses crucial symptoms such as suicidal ideation, a risk factor for work-related suicide. The construct of depression, which is deeply anchored in the ODI, has been and continues to be highly researched. A diagnosis of depression has consensual criteria, such as the widely employed criteria in the DSM-5, which contrasts sharply with Rotenstein et al.'s (34) finding that researchers have employed 142 different unique categorizations of burnout.

In contrast to other depressive symptom scales (e.g., the CES-D), the ODI explicitly asks respondents whether they attribute each depressive symptom to their job (e.g., “My experience at work made me feel like a failure”). The instrument has a protocol to help rule out symptoms attributed to nonwork sources (e.g., a conflictual spousal relationship) or a source the respondent cannot identify. The ODI can be used in two different ways. First, it can quantify work-related depressive symptoms along a continuum of severity. Second, the instrument produces provisional diagnoses of work-related depression. The ODI is currently available in English, French, and Spanish (see the Supplemental Material).

Because the ODI can help identify workers with clinically significant job-related distress, occupational health specialists (e.g., physicians, psychologists) can direct affected workers to treatment. We also recommend that specialists investigate workplace stressors that contribute to distress and take steps to ameliorate the stressful working conditions. The ODI can also help epidemiologists estimate the prevalence of job-related depression in organizations and occupational sectors. Because the instrument is brief, it reduces the burden on the respondent completing it. Unlike the MBI, the ODI is available at no cost.

Compared to the ODI, MBI items manifest redundancy (e.g., “Working with people all day is really a strain for me;” “Working with people directly puts too much stress on me)”, artificially inflating the instrument's reliability. The ODI does not exhibit this limitation because each of its nine items focuses on a specific DSM-5 symptom of major depression. Item wording shows no explicit redundancy—yet the ODI has optimal reliability.

Because the ODI deliberately assesses symptoms the individual attributes to perceived job stress, we recommend *against* using the instrument in cross-sectional research linking the ODI to perceived job-stressor measures. We recommend research that employs longitudinal designs or that links objective measures of job adversity to the ODI.

We created the ODI for two practical purposes: (1) to help occupational health specialists identify suffering workers and take the appropriate steps to remediate the problem and (2) to aid epidemiologists in estimating the prevalence of job-related depression such that appropriate public health policies can be established. A corollary to those two practical purposes is that the ODI can help specialists identify depressogenic organizations. For example, if within a given economic sector, the prevalence of occupational depression is, say, 2.5% but in one organization the prevalence is 25%, problematic working conditions are likely to plague that organization. The ODI would be the sentinel that signals occupational health specialists to take action to help troubled workers and improve workplaces.

## Data Availability Statement

The original contributions presented in the study are included in the article/Supplementary Material, further inquiries can be directed to the corresponding author.

## Author Contributions

ISS wrote the initial draft of the paper. RB edited the draft and added to it. The draft went back and forth between ISS and RB several times during which the two authors iteratively shaped the paper and settled on its final form. Both authors contributed to the article and approved the submitted version.

## Conflict of Interest

The authors declare that the research was conducted in the absence of any commercial or financial relationships that could be construed as a potential conflict of interest.

## Publisher's Note

All claims expressed in this article are solely those of the authors and do not necessarily represent those of their affiliated organizations, or those of the publisher, the editors and the reviewers. Any product that may be evaluated in this article, or claim that may be made by its manufacturer, is not guaranteed or endorsed by the publisher.
