# Supplementary material for: From Burnout to Occupational Depression: Recent Developments in Research on Job-Related Distress and Occupational Health
Source: Front Public Health. 2021 Dec 10;9:796401. doi: 10.3389/fpubh.2021.796401 (PMC8702721; doi:10.3389/fpubh.2021.796401)
Supplement: Supplementary file 1 [file Data_Sheet_1.PDF]

SUPPLEMENTAL MATERIAL

THE OCCUPATIONAL DEPRESSION INVENTORY IN THREE LANGUAGES

ENGLISH

FRENCH

SPANISH

OCCUPATIONAL DEPRESSION INVENTORY (ODI)

PRELIMINARY INSTRUCTIONS TO RESPONDENTS

The following statements concern the impact your work could have had on you.

Please read each statement and indicate how often you experienced the problems mentioned over the PAST TWO WEEKS. Use the scale provided to respond:

0 = never or almost never

1 = a few days only

2 = more than half the days

3 = nearly every day

Here is an example:

"I felt anxious because of my job."

• If you did NOT feel anxious because of your job, select **0**.

• If you felt anxious for reasons that you consider UNCONNECTED TO YOUR JOB (personal problems, marital problems, family problems, health problems, etc.), select **0** as well.

• If you felt anxious but don't know why, again select **0**.

• If it is clear for you that YOUR JOB caused you to feel anxious, select **1**, **2** or **3** to indicate how often that happened.

You can now complete the questionnaire.

OCCUPATIONAL DEPRESSION INVENTORY (ODI)

Patient name: ……………………………………………………… Date: …………………………………………

Indicate how often you experienced the problems mentioned below over the past two weeks.	*Never or almost never*	*A few days only*	*More than half the days*	*Nearly every day*
1. My work was so stressful that I could not enjoy the things that I usually like doing.	**0**	**1**	**2**	**3**
2. I felt depressed because of my job.	**0**	**1**	**2**	**3**
3. The stress of my job caused me to have sleep problems (I had difficulties falling asleep or staying asleep, or I slept much more than usual).	**0**	**1**	**2**	**3**
4. I felt exhausted because of my work.	**0**	**1**	**2**	**3**
5. I felt my appetite was disturbed because of the stress of my job (I lost my appetite, or the opposite, I ate too much).	**0**	**1**	**2**	**3**
6. My experience at work made me feel like a failure.	**0**	**1**	**2**	**3**
7. My job stressed me so much that I had trouble focusing on what I was doing (e.g., reading a newspaper article) or thinking clearly (e.g., to make decisions).	**0**	**1**	**2**	**3**
8. As a result of job stress, I felt restless, or the opposite, noticeably slowed down—for example, in the way I moved or spoke.	**0**	**1**	**2**	**3**
9. I thought that I'd rather be dead than continue in this job.	**0**	**1**	**2**	**3**

TOTAL SCORE: ………………………………………………

If you have encountered at least some of the problems mentioned above, do these problems lead you to consider leaving your current job or position?

☐ Yes ☐ No ☐ I don't know

Bianchi, R., & Schonfeld, I. S. (2020). The Occupational Depression Inventory: A new tool for clinicians and epidemiologists. *Journal of Psychosomatic Research, 138*, 110249. https://doi.org/10.1016/j.jpsychores.2020.110249

INVENTAIRE DE DÉPRESSION PROFESSIONNELLE (IDP)

INSTRUCTIONS PRÉLIMINAIRES AUX RÉPONDANTS

Les propositions suivantes concernent l'impact que votre travail aurait pu avoir sur vous.

Veuillez lire chaque proposition et indiquer à quelle fréquence vous avez rencontré les problèmes mentionnés au cours des DEUX DERNIÈRES SEMAINES. Utilisez l'échelle fournie pour répondre :

0 = jamais ou presque jamais

1 = quelques jours seulement

2 = plus de la moitié du temps

3 = tous les jours ou presque

Voici un exemple :

« Je me suis senti(e) anxieux/se à cause de mon travail. »

• Si vous NE vous êtes PAS senti(e) anxieux/se à cause de votre travail, sélectionnez **0**.

• Si vous vous êtes senti(e) anxieux/se pour des raisons qui sont selon vous SANS LIEN AVEC VOTRE TRAVAIL (problèmes personnels, conjugaux, familiaux, de santé, etc.), sélectionnez également **0**.

• Si vous vous êtes senti(e) anxieux/se mais ne savez pas pourquoi, de nouveau sélectionnez **0**.

• S'il est clair pour vous que VOTRE TRAVAIL vous a rendu anxieux/se, sélectionnez **1**, **2** ou **3** pour indiquer à quelle fréquence cela s'est produit.

Vous pouvez maintenant répondre au questionnaire.

INVENTAIRE DE DÉPRESSION PROFESSIONNELLE (IDP)

Nom du patient : ……………………………………………… Date : ………………………………………………

**Indiquez à quelle fréquence vous avez fait l'expérience des problèmes évoqués ci-dessous au cours des deux dernières semaines.**	*Jamais ou presque jamais*	*Quelques jours seulement*	*Plus de la moitié du temps*	*Tous les jours ou presque*
1. Mon travail a été si stressant que je n'arrivais pas à profiter des choses qui d'habitude me font plaisir.	**0**	**1**	**2**	**3**
2. Je me suis senti(e) déprimé(e) à cause de mon travail.	**0**	**1**	**2**	**3**
3. J'ai senti que mon sommeil était perturbé à cause de problèmes liés à mon travail (j'ai éprouvé des difficultés à m'endormir ou à rester endormi(e), ou au contraire j'ai dormi beaucoup plus que d'habitude).	**0**	**1**	**2**	**3**
4. Je me suis senti(e) épuisé(e) à cause de mon travail.	**0**	**1**	**2**	**3**
5. J'ai senti que mon appétit était perturbé à cause du stress lié à mon travail (j'ai perdu l'appétit, ou au contraire j'ai mangé beaucoup plus que d'habitude).	**0**	**1**	**2**	**3**
6. Je me suis senti(e) en échec personnel à cause de mon travail.	**0**	**1**	**2**	**3**
7. Mon travail m'a stressé(e) à tel point que j'ai eu du mal à me concentrer sur ce que je faisais (par exemple, lire la presse) ou à penser clairement (par exemple, pour prendre des décisions).	**0**	**1**	**2**	**3**
8. À cause du stress lié à mon travail, je me suis senti(e) sensiblement agité(e), ou au contraire sensiblement ralenti(e) – par exemple dans ma façon de bouger ou de parler.	**0**	**1**	**2**	**3**
9. Je me suis dit que je préférerais être mort(e) plutôt que de continuer dans ce travail.	**0**	**1**	**2**	**3**

SCORE TOTAL : ……………………………………………………..

Si vous avez rencontré au moins certains des problèmes évoqués ci-dessus, ces problèmes vous amènent-ils à envisager de quitter votre emploi ou poste actuel ?

☐ Oui ☐ Non ☐ Je ne sais pas

Bianchi, R., & Schonfeld, I. S. (2020). The Occupational Depression Inventory: A new tool for clinicians and epidemiologists. *Journal of Psychosomatic Research, 138*, 110249. https://doi.org/10.1016/j.jpsychores.2020.110249

INVENTARIO DE DEPRESIÓN PROFESIONAL (IDP)

INSTRUCCIONES PRELIMINARES PARA LOS/AS ENCUESTADOS/AS

Las siguientes afirmaciones aluden al posible impacto que su trabajo podría tener sobre usted.

Lea cada afirmación e indique con qué frecuencia se ha encontrado con los problemas mencionados en las ÚLTIMAS DOS SEMANAS. Utilice la escala proporcionada aquí para responder:

0 = nunca o casi nunca

1 = sólo unos días

2 = más de la mitad de las veces

3 = todos los días o casi todos

Aquí tiene un ejemplo:

«Me he sentido ansioso/a por causa de mi trabajo»

• Si no se ha sentido ansioso/a por su trabajo, seleccione **0**.

• Si se ha sentido ansioso/a por razones que usted considera QUE NO TIENE RELACIÓN CON SU TRABAJO (problemas personales, conyugales, familiares, de salud, etc.), seleccione igualmente **0**.

• Si se ha sentido ansioso/a pero no sabe porque, seleccione de nuevo **0**.

• Si usted tiene claro que SU TRABAJO le provoca ansiedad, seleccione **1**, **2** o **3** para indicar la frecuencia con la que se ha producido.

Ahora puede contestar al cuestionario.

INVENTARIO DE DEPRESIÓN PROFESIONAL (IDP)

Nombre del paciente: …………………………………………… Fecha: ……………………………………………..

**Indique con que frecuencia ha experimentado los problemas que se señalan a continuación en el transcurso de las dos últimas semanas.**	*Nunca o casi nunca*	*Sólo unos días*	*Más de la mitad de las veces*	*Todos los días o casi todos*
1. Mi trabajo ha sido tan estresante que no he podido disfrutar de las cosas que habitualmente me producen satisfacción o placer.	**0**	**1**	**2**	**3**
2. Me he sentido deprimido/a por causa del trabajo.	**0**	**1**	**2**	**3**
3. He padecido alteraciones del sueño por el estrés vivido en mi trabajo (he experimentado dificultades para dormirme o para quedarme dormido/a o a permanecer dormido/a, o por el contrario he dormido mucho más de lo habitual).	**0**	**1**	**2**	**3**
4. Me he sentido agotado/a por causa del trabajo.	**0**	**1**	**2**	**3**
5. He experimentado alteraciones del apetito por causa del estrés en mi trabajo (he perdido el apetito, o por el contrario he comido mucho más de lo habitual).	**0**	**1**	**2**	**3**
6. Me he sentido fracasado/a personalmente por causa de mi trabajo.	**0**	**1**	**2**	**3**
7. Mi trabajo me ha estresado a tal punto que he tenido dificultades para concentrarme sobre lo que estaba haciendo (por ejemplo, leer la prensa) o a pensar con claridad (por ejemplo, para tomar decisiones).	**0**	**1**	**2**	**3**
8. A causa del estrés relacionado con mi trabajo, me he sentido muy agitada/o, o por el contrario muy ralentizada/o (por ejemplo, en la forma de moverme o de hablar).	**0**	**1**	**2**	**3**
9. He llegado a pensar que preferiría estar muerta/o en lugar de seguir trabajando en este trabajo.	**0**	**1**	**2**	**3**

PUNTUACIÓN TOTAL: ………………………………………………………...

Si usted se ha identificado con algunos de los problemas sugeridos anteriormente, ¿estos problemas le están haciéndose plantear la posibilidad de dejar su trabajo o puesto actual?

☐ Sí ☐ No ☐ No lo sé

Bianchi, R., & Schonfeld, I. S. (2020). The Occupational Depression Inventory: A new tool for clinicians and epidemiologists. *Journal of Psychosomatic Research, 138*, 110249. https://doi.org/10.1016/j.jpsychores.2020.110249
